# Histone Deacetylase 1/Sp1/MicroRNA-200b Signaling Accounts for Maintenance of Cancer Stem-Like Cells in Human Lung Adenocarcinoma

**DOI:** 10.1371/journal.pone.0109578

**Published:** 2014-10-03

**Authors:** Dong-Qin Chen, Jia-Yuan Huang, Bing Feng, Ban-Zhou Pan, Wei De, Rui Wang, Long-Bang Chen

**Affiliations:** 1 Department of Medical Oncology, Jinling Hospital, School of Medicine, Nanjing University, Nanjing, Jiangsu, P. R. China; 2 Department of Biochemistry and Molecular Biology, Nanjing Medical University, Nanjing, P. R. China; H.Lee Moffitt Cancer Center & Research Institute, United States of America

## Abstract

The presence of cancer stem-like cells (CSCs) is one of the mechanisms responsible for chemoresistance that has been a major hindrance towards lung adenocarcinoma (LAD) treatment. Recently, we have identified microRNA (miR)-200b as a key regulator of chemoresistance in human docetaxel-resistant LAD cells. However, whether miR-200b has effects on regulating CSCs remains largely unclear and needs to be further elucidated. Here, we showed that miR-200b was significantly downregulated in CD133^+^/CD326^+^ cells that exhibited properties of CSCs derived from docetaxel-resistant LAD cells. Also, restoration of miR-200b could inhibit maintenance and reverse chemoresistance of CSCs. Furthermore, suppressor of zeste-12 (Suz-12) was identified as a direct and functional target of miR-200b, and silencing of Suz-12 phenocopied the effects of miR-200b on CSCs. Additionally, overexpression of histone deacetylase (HDAC) 1 was identified as a pivotal mechanism responsible for miR-200b repression in CSCs through a specificity protein (Sp) 1-dependent mechanism, and restoration of miR-200b by HDAC1 repression significantly suppressed CSCs formation and reversed chemoresistance of CSCs by regulating Suz-12-E-cadherin signaling. Also, downregulation of HDAC1 or upregulation of miR-200b reduced the *in vivo* tumorigenicity of CSCs. Finally, Suz-12 was inversely correlated with miR-200b, positively correlated with HDAC1 and up-regulated in docetaxel-resistant LAD tissues compared with docetaxel-sensitive tissues. Taken together, the HDAC1/miR-200b/Suz-12-E-cadherin signaling might account for maintenance of CSCs and formation of chemoresistant phenotype in docetaxel-resistant LAD cells.

## Introduction

Lung cancer accounts for the most cancer-related mortalities in both women and men worldwide [1]. Chemotherapy is an important component of the first-line therapies for lung adenocarcinoma (LAD) that constitutes the most common histological form of lung cancer. However, chemoresistance represents a predominant obstacle towards chemotherapeutic treatment of LAD, which leads to poor prognosis of the patients. Thus, exploring the possible molecular mechanisms involved in chemoresistance has become a key issue in clinical treatment of human LAD.

Cancer stem-like cells (CSCs) or tumor initiating stem cells are a sub-population of tumor cells and play pivotal roles in cancer initiation, progression, recurrence and chemoresistance [2]–[5]. CSCs, derived from both CSCs and non-CSCs, give rise to tumors through self-renewal and are able to differentiate into multiple cell types [6]–[9]. Many cancer therapies including chemotherapies that kill the bulk of cancer cells, may ultimately fail as they do not eliminate CSCs that then cause a relapse of tumors [10]. Recently, it has been firmly established that CSCs are linked to epithelial-mesenchymal transition (EMT), metastasis, drug resistance, progression and relapse of lung cancer [11]–[15]. As a result, exploitation of the specific therapies targeting at CSCs has been a crucial issue in chemotherapeutic treatment of lung cancer. MicroRNAs (miRNAs) silence gene expression by binding to the 3′-untranslated region of the target genes and have been reported to regulate CSCs self-renewal, tumorigenicity, metastasis, and chemoresistance in many human cancers [2], [7], [16]. For example, miR-34a repression causes colon CSCs to perform asymmetric cell division and promotes daughter cells to remain colon CSCs by regulating Notch signaling. Upregulated miR-143 in CSCs differentiation promotes prostate cancer cells metastasis by modulating FNDC3B expression. MiR-21 regulates EMT phenotype and hypoxia-inducible factor-1α expression in third-sphere forming breast cancer stem cell-like cells. MiR-200b, an important member of miR-200 families, is located at miR-200b/c/429 gene cluster, acts as a tumor suppressor in a variety of human solid tumors and has the capability of inhibiting CSCs growth and reversing the EMT phenotype of CSCs [17], [18]. Recently, we have identified miR-200b as a key regulator of chemoresistance and restoration of miR-200b significantly reverses chemoresistance of docetaxel (DTX)-resistant LAD cells by inducing cell cycle arrest and apoptosis enhancement [19]. However, whether miR-200b regulates CSCs derived from docetaxel-resistant LAD cells is still poorly understood and needs to be further elucidated.

In this study, we first show that miR-200b functions as a tumor suppressor both *in vitro* and in *vivo* in CSCs that are originated from human docetaxel-resistant LAD cells. Also, we identify HDAC1 as a specific regulator involved in silencing of miR-200b through a Sp1-dependent mechanism, and restoration of miR-200b mediated by HDAC1 repression significantly suppresses maintenance of CSCs and reverses chemoresistance of CSCs by regulating Suz-12-E-cadherin signaling. To the best of our knowledge, there have been no reports about HDAC1/miR-200b/Suz-12/E-cadherin regulatory network in regulating CSCs maintenance and chemoresistance in human LAD cells and the current work will provide a novel strategy for reversing chemoresistance of human LAD.

## Materials and Methods

### Ethics statement

This study was approved by the research ethics committee of Jinling Hospital of NanJing University (Permit Number: 12-027) and was performed in compliance with the Helsinki Declaration. All animals were housed under specific pathogen-free conditions. All experimental procedures were performed in accordance with the Jinling Hospital of Nanjing University Guide for the Care and Use of Laboratory Animals.

### MicroBeads sorting

Briefly, 3.0×10^7^ single cells were passed through 30 µm nylon mesh and centrifuged at 300×g for 10 minutes. The supernatant was then aspirated. Then, 300 µl buffer and 100 µl FcR reagent were added and mixed. 100 µl CD326 Microbeads were added, mixed well and incubated for 30 minutes in the refrigerator (2–8°C). Cells were then washed by adding 2 mL of buffer and centrifuged at 300×g for 10 minutes. The supernatant was then aspirated. Cells were resuspended with 500 µl of buffer. Then the cell suspension was applied onto the separator column placed in the magnetic field. The flow-through containing unlabeled cells was collected. Then the column was washed and removed from the separator and placed on a suitable collection tube. Then, the appropriate amount of buffer was pipetted onto the column and the flow-through containing CD326 cells was collected. At last, the appropriate number of CD326^+^ cells was then sorted with CD133 Microbeads for the enrichment of CD133^+^/CD326^+^ cells as described above. The CD133^+^/CD326^+^ CSCs were then cultured in serum-free CSCs-culturing medium: Dulbecco's modified eagle medium (DMEM)/F12 medium containing 0.4% BSA, 2% B27, 4 mg/ml insulin and 40 ng/ml EGF.

### Cells and culture conditions

The human LAD cell lines (SPC-A1 and H1299) were purchased from the Tumor Cell Bank of Chinese Academy of Medical Science (Shanghai, China). The docetaxel-resistant LAD cells were prepared as described in our previous work and preserved in 50.0µg/L docetaxel [19]. Cells were cultured as described in our previous work [20]. The CD133^+^/CD326^+^ cells were sorted from the docetaxel-resistant LAD cells by CD133 and CD326 MicroBeads (Miltenyi Biotec) according to the manufacturer's instructions.

### Patient samples

The present work was approved by the Ethic Committee of our hospital and written informed consent was obtained from all of the patients. LAD tissues were gathered from patients with LAD at advanced stage in our hospital from September 2006 to December 2008. The patients met all of the following criteria: histological diagnosis of primary LAD with at least one measurable lesion; clinical stage IIIB-IV; first-line chemotherapy either with docetaxel 75 mg/m^2^ and cisplatin 100 mg/m^2^ or docetaxel 75 mg/m^2^ and carboplatin area under the curve 6 mg/mL/min administered every 3 weeks for a maximum of 5 cycles. Tissue samples were then divided into “sensitive” (complete or partial response) and “insensitive” (stable or progressive disease) groups basing on the patient's responses assessed by medical image analysis and detection of serum tumor markers after 4 or 5 cycles of docetaxel-based chemotherapy.

### Plasmids and cell transfections

Recent studies have reported that miR-200b has two functional promoters, located ∼4 kb and ∼2 kb upstream of the 5′-stemloop, respectively [21]. Then, the two core regions, located chr1:1087797-1088137 (341 bp) and chr1:1089316-1090354 (1039 bp), respectively (UCSC Genome Browser, March 2006) were amplified by PCR and cloned into pGL3-basic vector (Promega) with KpnI and HindIII sites, named (pGL3) promoter-1 and promoter-2. Furthermore, multiple Sp1 binding sites were observed in the two core regions. And, the Sp1 binding sites located chr1:1087926-1087932, chr1:1088073-1088079 and chr1:1089779-1089785, respectively. Then, Sp1-binding-site-directed mutant reporter vectors were constructed by mutation PCR. Luciferase reporter containing wild type 3′-UTR of Suz-12(pLUC/Suz-12/3′-UTR-wt) in which the nucleotides of the Suz-12-3′-UTR complementary to miR-200b were inserted into the pLUC vector, and we also generated a mutant reporter (pLUC/Suz-12/3′-UTR-mut), in which the first six nucleotides in the miR-200b seed region complementary sites were mutated. All of the primer sequences were listed in **Table S1.** MiR-200b expression plasmid (pcDNA/miR-200b), the mock pcDNA-negative control (pcDNA/miR-NC), miR-200b inhibitor (Anti-miR-200b) and nonspecific miRNA control (Anti-miR-NC) were used as our previous described [18]. HDAC1-shRNA (or control-shRNA), Sp1-shRNA (or control-shRNA) vectors were obtained from GenePharma. Suz-12 expression plasmid (pcDNA/Suz-12) was constructed by subcloning Suz-12 into the pcDNA 3.0 vector (Invitrogen) by PCR with the primers listed in **Table S2**. All of the vectors were confirmed by DNA sequencing. Cells were transfected with Lipofectamine 2000 (Invitrogen) according to the manufacturer's instructions.

### Flow cytometric analysis of CD133^+^/CD326^+^ cells

Flow cytometric analysis of CD133^+^/CD326^+^ cells was performed with CD133-PE and CD326-FITC antibodies (Miltenyi Biotec) according to the manufacturer's instructions. Briefly, approximately 1.0×10^6^ cells were centrifuged at 300×g for 10 minutes. The supernatant was then discarded. The cells were resuspended with 80 µL of buffer. 20 µL of FcR Blocking Reagent was then added into the buffer. 10 µL of the CD133/CD326 antibody was added, mixed well and incubated for 10 minutes in the dark in the refrigerator (2–8°C). Next, the cells were washed by adding 1–2 mL of buffer and centrifuge at 300×g for 10 minutes. The supernatant was then aspirated. At last, the cells were resuspended in 400µL of buffer for analysis by flow cytometry.

### Mammosphere formation assay

Mammosphere formation assay was performed to assess the ability of CSC self-renewal. Mammosphere formation assay was conducted by plating single-cell suspensions (1000 cells/well) into ultra-low adherent 6-well plates. Then, the cells were grown in serum-free CSCs-culturing medium and supplemented with the medium every 3 days. After 7 days of incubation, the plates were analyzed for measurement of mammosphere numbers.

### Western blotting assay

The primary antibodies against E-cadherin, Sox-2 and Oct-4 (Abcam, HongKong), Bmi-1, HDAC1, Suz-12 (Cell Signaling Technologies) and GAPDH (Santa Cruz Biotechnology) were used in this study. GAPDH was used as an internal control. Total protein lysate was separated by 10% sodium dodecyl sulfate-polyacrylamide gel electrophoresis (SDS-PAGE). The proteins were then transferred onto polyvinylidene fluoride (PVDF) membranes (Millipore). Next, immunoblotting was performed and visualized with a chemiluminescence kit (Thermo Scientific).

### Real-time quantitative reverse-transcription polymerase chain reaction (qRT-PCR) assay

Total RNA was extracted with TRIzol reagent (Takara). Reverse transcription was performed with PrimeScript RT reagent Kit (Takara) basing on the manufacturer's instructions. qRT-PCR was performed with SYBR PrimeScript RT-PCR Kits (Takara) according to the manufacturer's instructions. The miR-200b or mRNA levels were calculated with the 2^−△△Ct^ method using U6 rRNA or GAPDH mRNA as the reference genes. The expression levels were measured relative to the fold change of the control cells that was defined as 1.0. All experiments were conducted in triplicate. All of the primer pairs were listed in **Table S3**.

### Drug sensitivity and cell viability assay

Drug sensitivity and cell viability assay was measured with Cell Counting Kit-8 (CCK-8) assay (Dojindo) according to the manufacturer's instructions. For Drug sensitivity assay, 3×10^3^ cells were seeded into 96-well plates 24 hours after transfection. After attachment, freshly prepared docetaxel was then added at different final concentration. 48 hours later, 10 µL of CCK-8 was added and incubated for 4 hours at 37°C. Absorbance was then measured at 450 nm. For cell viability assay, 2.0×10^3^ cells were seeded into 96-well plates 24 hours after transfection with the indicated vectors. 12, 24 or 48 hours later, 10 µL of CCK-8 was added and incubated for 4 hours at 37°C. Absorbance was then measured at 450 nm. All experiment were performed in triplicate and repeated at least three times.

### Luciferase reporter assay

Approximately 2.0×10^3^/well CSCs were seeded into 96-well plates and then cotransfected with the specific luciferase reporter plasmids, miR-NC or miR-200b. Renila-TK plasmid (Promega) was cotransfected into all samples. 48 hours after transfection, luciferase activities were measured with Dual-Luciferase Reporter Assay kits (Promega). The luciferase activities were normalized by renilla luciferase activities. The data were relative to the fold change of the corresponding control groups that was defined as 1.0. All experiments were performed in triplicate.

### Co-immunoprecipitation assay

The Co-immunoprecipitation assay was performed using Co-Immunoprecipitation Kits (Thermo scientific Pierce) basing to the manufacturer's instructions. Briefly, 75µg of affinity-purified antibodies (Sp1, HDAC1, Cell Signaling Technologies) were coupled into the spin columns. Cell lysate was then prepared and pre-cleared with the control agarose resins. Subsequently, the cell lysate was co-immunoprecipitated and eluted. Finally, the samples were measured by western blotting assay.

### Chromatin immunoprecipitation (ChIP) assay

ChIP assay was performed with Immunoprecipitation Assay Kits (Millipore) according to the manufacturer's instructions. Briefly, cells were cross-linked with 1% formaldehyde for 10 min at 37°C. The cells were then resuspended in 200 µl of lysis buffer and incubated for 10 minutes on ice. The lysate was sheared to lengths between 200 and 1000 base pairs by sonication. The supernatant was pre-cleared with a Salmon Sperm DNA/Protein A Agarose-50% Slurry. The recovered supernatant was incubated with Suz-12 immunoprecipitating antibody (Abcam), Histone H3 trimethyl-lysine 27(H3-trimethyl-k27) immunoprecipitating antibody (Millipore), Acetyl-Histone H3 (Lys9) (Millipore), or an isotype control IgG overnight at 4°C with rotation. Then, the antibody/DNA complex was collected using Salmon Sperm DNA/Protein A Agarose Slurry for one hour at 4°C with rotation. The complex was eluted by elution buffer. Then, the crosslinks were reversed with 5 M NaCl heating at 65°C for 4 hours. The DNA samples were then purified and measured by qRT-PCR. The primers were listed in **Table S4**.

### Xenograft transplantation

BALB/c athymic nude mice (Male, SPF, 4–6 weeks) were provided by the department of experiment animal center of our hospital. And the in-vivo study was ethically approved and performed according to the institutional guidelines. To perform tumorigenicity in nude mice, CSCs from SPC-A1/DTX cells stably transfected with miR-200b, miR-NC, sh-control or sh-HDAC1#2 vectors were subcutaneously injected into the right flank of nude mice. Tumors were harvested while they were palpable. To investigate the effect of miR-200b expression or HDAC1 repression on *in vivo* regulation of LAD cells, 3.0×10^6^ H1299/DTX cells stably transfected with sh-control, sh-HDAC1, miR-NC or miR-200b vectors, were subcutaneously transplanted into the right side of the posterior flank of nude mice. Tumor volumes were measured as previous described [19]. While the average tumor dimension reached about 50 mm^3^, a concentration of 1.0 mg/kg docetaxel (DTX), one dose every other day, with three doses in total, was administered through intraperitoneal injection [19]. After 5 weeks, all mice were sacrificed, and tumor tissues were used for the subsequent studies.

### Statistical analysis

All statistical analysis was performed SPSS program version 17.0 (SPSS Inc., Chicago, IL, USA). Data are presented as mean ± standard error of the mean, and error bars are representative of at least three independent experiments. The mean values of two groups were compared by Student's *t* test. Differences between multiple groups were analyzed by one-way ANOVA analysis. All *P* values <0.05 were considered statistically significant.

## Results

### CD133^+^/CD326^+^ cells exhibit properties of CSCs

**Figure 1:**
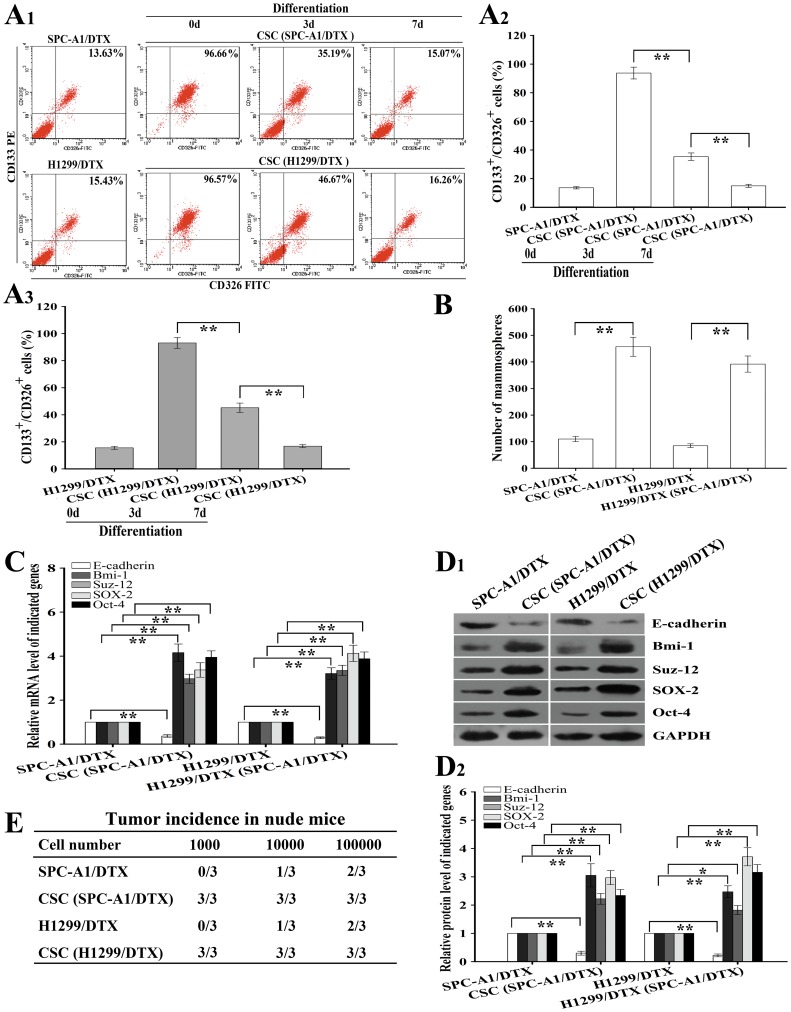
Properties of CSCs obtained from docetaxel-resistant human lung adenocarcinoma cells (SPC-A1/DTX and H1299/DTX). (A_1_, A_2_, A_3_) Percentage of CD133^+^/CD326^+^ CSCs as analyzed by Flow cytometry at the indicated time after cultivating CD133^+^/CD326^+^ CSCs (acquired by sorting SPC-A1/DTX and H1299/DTX cells, respectively) under differentiation conditions. (B) Mammosphere forming ability of the indicated cells (1000 cells) after 7 days under CSC-cultivating conditions. (C) qRT-PCR detection of relative mRNA levels of the CSC-related markers in the indicated cells. Data was calculated with the 2^−△△Ct^ method using GAPDH RNA as the reference gene. The expression level was measured relative to the fold change of the parental cells groups that were defined as 1.0. (D_1_, D_2_) The protein levels of the CSC-related markers in the indicated cells. GAPDH was used as an internal control. (E) Tumor incidence in nude mice that were subcutaneously injected with the indicated cells. Data were presented as mean ± SD of at least three independent experiments. *p<0.05, **p<0.01.

To better understand the underlying mechanisms responsible for chemoresistance in LAD, CD133^+^/CD326^+^ cells were sorted from the docetaxel-resistant LAD cells by CD133 and CD326 MicroBeads. The purity of CD133^+^/CD326^+^ cells sorted from SPC-A1/DTX and H1299/DTX was 93.13%±4.06% and 92.74%±3.12%, respectively, and the CD133^+^/CD326^+^ cells could differentiate to the docetaxel-resistant LAD cells while under differentiation condition (**Figure 1A** **_1_, A_2_ and A_3_**). Then, we determined whether the CD133^+^/CD326^+^ cells displayed properties of CSCs. First, we analyzed the mammosphere forming ability of the CD133^+^/CD326^+^ cells, and the data showed that the number of mammosphere in CD133^+^/CD326^+^ cells was significantly more than that in the corresponding docetaxel-resistant LAD cells (**Figure 1B**). Also, the CD133^+^/CD326^+^ cells expressed higher expression levels of stem cell markers (such as Sox-2, Oct-4, Suz-12, and Bmi-1) than the corresponding docetaxel-resistant LAD cells (**Figure 1C, 1D** **_1_ and D_2_**). Second, we analyzed the tumorigenic capacity of the CD133^+^/CD326^+^ cells, and then about 1,000∼100,000 CD133^+^/CD326^+^ cells or the corresponding docetaxel-resistant LAD cells were subcutaneously inoculated into nude mice. 1,000 CD133^+^/CD326^+^ cells were sufficient to form tumors in all of the nude mice, however, up to 1.0×10^5^ docetaxel-resistant LAD cells was unable to successfully form tumors in each nude mice, which indicated that the CD133^+^/CD326^+^ cells possessed higher capability of tumorigenicity than the parental docetaxel-resistant LAD cells (**Figure 1E**). Therefore, the CD133^+^/CD326^+^ subpopulation cells displayed properties of CSCs.

### MiR-200b inhibits CSCs formation and reverses chemoresistance of CSCs

**Figure 2:**
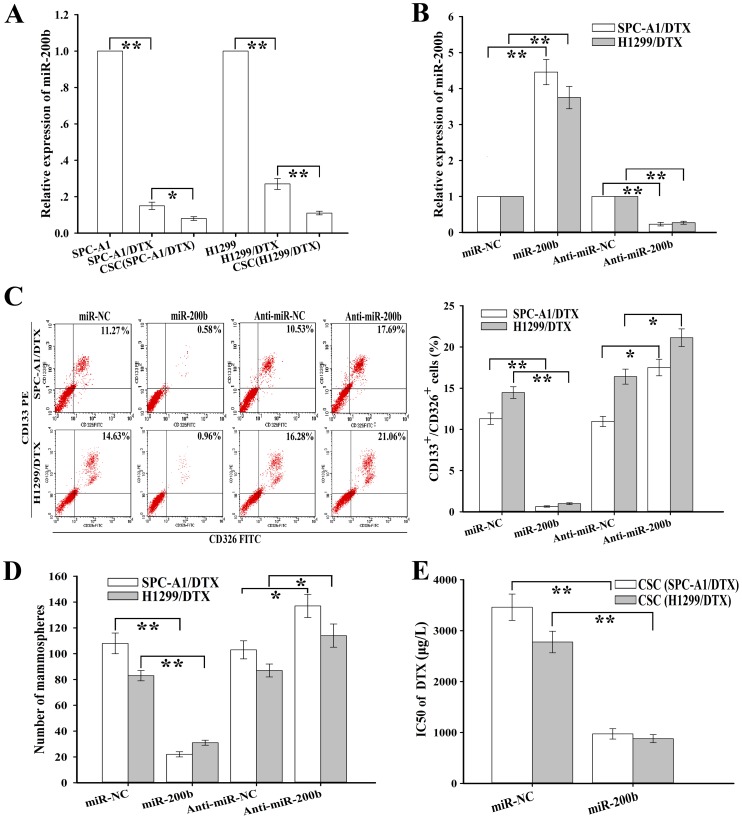
MiR-200b inhibits CSCs growth and reverses chemoresistance of CSCs. (**A**) qRT-PCR detection of miR-200b in CSCs. Data were normalized to U6 rRNA and determined relative to the corresponding parental cell groups. (**B**) qRT-PCR detection of relative mRNA level of miR-200b in docetaxel-resistant LAD cells after transfection with the indicated vectors. Data were normalized to U6 rRNA and determined relative to the corresponding control groups. (**C**) Percentage of CD133^+^/CD326^+^ CSCs as analyzed by flow cytometry after transfection with the indicated vectors. (**D**) Mammosphere forming ability of the indicated cells (1000 cells) after transfection of the indicated vectors. (**E**) Cell viability were measured by Cell Counting Kit-8 (CCK-8) assay. Data were presented as mean ± SD of at least three independent experiments. *p<0.05, **p<0.01.

Previously, we have shown that upregulation of miR-200b can reverse chemoresistance of the docetaxel-resistant LAD cells by inhibiting cell growth, inducing cell cycle arrest and enhancing apoptosis. Then, we performed qRT-PCR assay to detect the expression of miR-200b in CSCs and the corresponding docetaxel-resistant LAD cells, and showed that the relative level of miR-200b in CSCs was lower than that in the docetaxel-resistant LAD cells (**Figure 2A**). To further investigate whether has miR-200b effect on CSCs in the docetaxel-resistant LAD cells, pcDNA/miR-200b (or pcDNA/miR-NC) or anti-miR-200b (or anti-miR-NC) was stably or transiently transfected into SPC-A1/DTX or H1299/DTX cells. As shown in **Figure 2B**, the relative expression level of miR-200b was significantly upregulated in docetaxel-resistant LAD cells stably transfected with pcDNA/miR-200b, while its relative expression level was significantly downregulated in those cells transiently transfected with anti-miR-200b. Flow cytometry assay showed that upregulation of miR-200b significantly decreased the percentage of CD133^+^/CD326^+^ CSCs growth in the docetaxel-resistant LAD cells and downregulation of miR-200b had the opposite effect (**Figure 2C**). The similar phenomenon was observed in mammosphere forming ability assay as well (**Figure 2D**). Meanwhile, restoration of miR-200b could lead to the decreased IC_50_ value of DTX in CSCs from SPC-A1/DTX or H1299/DTX cells (**Figure 2E**). These results indicated that miR-200b might play a critical role in CSCs growth and chemoresistance of human LAD.

### Suz-12 is upregulated in CSCs and identified as a functional target of miR-200b

**Figure 3:**
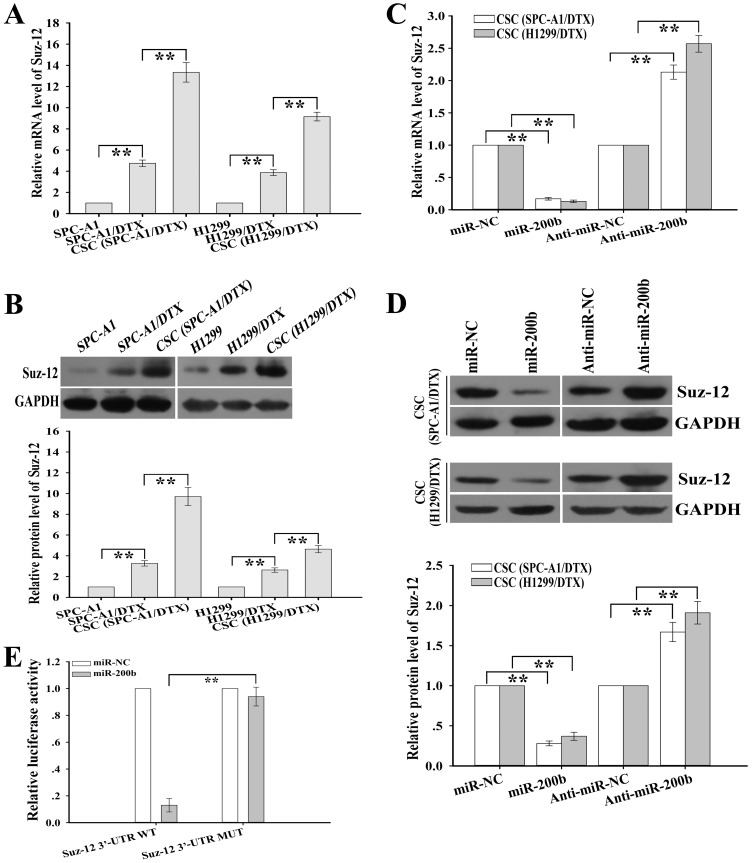
Identification of Suz-12 as a functional target of miR-200b in CSCs. (**A**) qRT-PCR detection of relative Suz-12 mRNA expression in the indicated cells. Data were normalized to GAPDH RNA and determined relative to the corresponding parental-cell groups. (**B**) The protein levels of Suz-12 as measured by Western blotting in the indicated cells. GAPDH was used as an internal control. (**C**) qRT-PCR detection of Suz-12 mRNA expression in CSCs after transfection of the indicated vectors. (**D**) Western blotting detection of Suz-12 protein expression in CSCs after transfection with the indicated vectors. GAPDH was used as an internal control. (**E**) Luciferase activity of the CSCs (SPC-A1/DTX) co-transfected with pcDNA/miR-200b (or pcDNA/miR-NC) or pLUC/Suz-12/3′-UTR-wt (or pLUC/Suz-12/3′-UTR-mut). Data were mean ± SD of at least three independent experiments. *p<0.05, **p<0.01.

Suz-12 is a component of the polycomb repressive complex 2 (PRC2) and is essential for PRC2-mediated gene silencing by generating trimethylation on lysine 27 residue of histone H3 (H3K27Me3). In CSCs generated from transformed breast epithelial cells (MCF-10A), Suz-12 has been confirmed as a target gene of miR-200b. However, whether miR-200b can target Suz-12 in the CSCs derived from the docetaxel-resistant LAD cells is unclear. qRT-PCR and Western blotting assays indicated that Suz-12 was up-regulated in CSCs compared with the corresponding docetaxel-resistant LAD cells at both transcriptional and translational levels (**Figure 3A and B**). Then, we analyzed the effects of miR-200b on the expression of Suz-12 in CSCs. Upregulation of miR-200b led to the decreased expression of Suz-12 in CSCs of SPC-A1/DTX and H1299/DTX cells, while silencing of miR-200b led to the increased expression of Suz-12 in those CSCs (**Figure 3C and D**). To obtain further direct evidence that Suz-12 was a target of miR-200b, we investigated the binding site of miR-200b in the 3′-UTR sequence of Suz-12 mRNA. We constructed a luciferase reporter (pLUC/Suz-12/3′-UTR-wt) in which the nucleotides of the Suz-12-3′-UTR complementary to miR-200b were inserted into the pLUC vector, and we also generated a mutant reporter (pLUC/Suz-12/3′-UTR-mut), in which the first six nucleotides in the miR-200b seed region complementary sites were mutated. Luciferase activity in the pLUC/Suz-12/3′-UTR-wt-transfected cells co-transfected with pcDNA/miR-200b was significantly decreased than that in the pLUC/Suz-12/3′-UTR-mut-transfected cells co-transfected with pcDNA/miR-200b (**Figure 3E**). These results suggested that Suz-12 might be a direct target of miR-200b in the CSCs derived from the docetaxel-resistant LAD cells.

**Figure 4:**
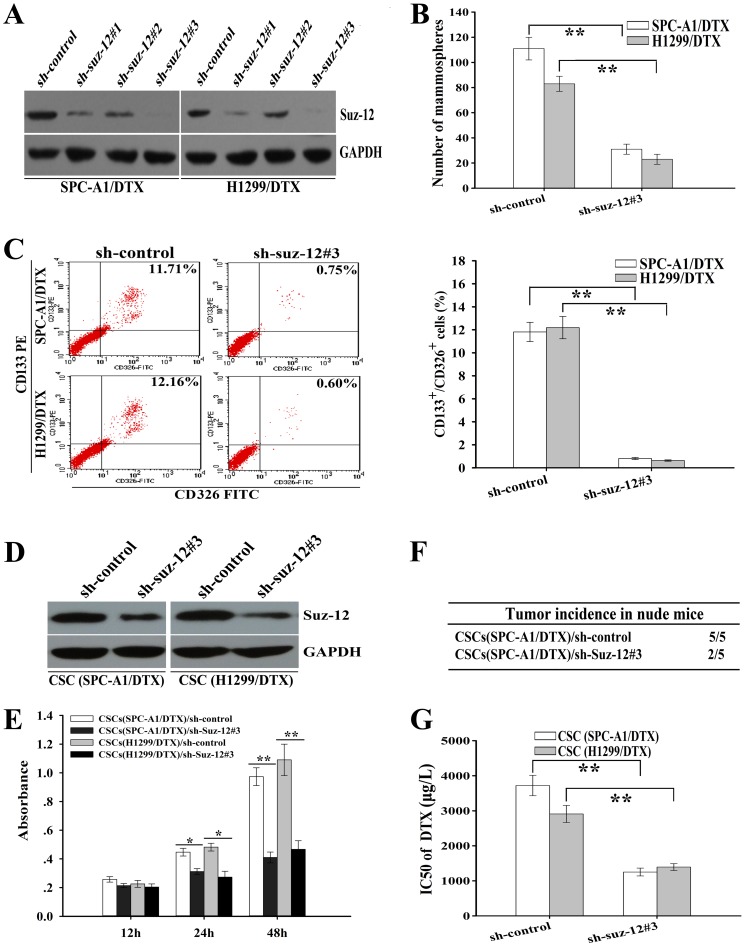
Silencing of Suz-12 inhibits CSCs growth and tumorigenicity and reverses chemoresistance of CSCs. (**A**) The protein level of Suz-12 in docetaxel-resistant LAD cells after transfection with the indicated sh-RNA vectors (sh-RNA-Suz12#1, sh-RNA-Suz12#2, and sh-RNA-Suz12#3). (**B**) Mammosphere forming ability of docetaxel-resistant LAD cells (1000 cells) after transfection of the indicated vectors. (**C**) Flow cytometry analysis of percentage of CD133^+^/CD326^+^ CSCs as analyzed by in docetaxel-resistant LAD cells after transfection of the indicated vectors. (**D**) The protein level of Suz-12 in CSCs after transfection with the sh-RNA-control or sh-RNA-Suz12#3 vectors. (**E**) CCK-8 analysis of cell viability of the CSCs transfected with the indicated vectors at the indicated time. (**F**) Effect of Suz-12 inhibition on *in vivo* tumorigenicity of the CSCs from SPC-A1/DTX cells. (**G**) Effect of Suz-12 inhibition on chemoresistance of CSCs. Cell viability was measured by CCK-8 assay. GAPDH was used as an internal control. Data were presented as mean ± SD of at least three independent experiments. *p<0.05, **p<0.01.

To further determine whether Suz-12 was involved in regulation of CSCs, Suz-12-shRNAs (Suz-12-shRNA#1, #2 or #3) were designed and transfected into docetaxel-resistant LAD cells and the inhibitory effect of Suz-12-shRNA3 was biggest (**Figure 4A**). Silencing of Suz-12 could significantly reduce the mammosphere forming ability of SPC-A1/DTX and H1299/DTX cells (**Figure 4B**). Also, the percentage of CD133^+^/CD326^+^ CSCs growth in Suz-12-shRNA3-transfected SPC-A1/DTX or H1299/DTX cells was significantly decreased than that in the control-shRNA-transfected cells (**Figure 4C**). Then, we further analyzed the effects of Suz-12 expression on the tumorigenicity and chemosensitivity of CSCs. First, we detected the protein expression of Suz-12 in CSCs from Suz-12-shRNA3 or control-shRNA-transfected SPC-A1/DTX and H1299/DTX cells, and showed that the expression level of Suz-12 protein in Suz-12-shRNA3-transfected cells was significantly lower than that in control-shRNA-transfected cells (**Figure 4D**). Functional analysis indicated that silencing of Suz-12 could significantly inhibit *in vitro* growth and *in vivo* tumorigenicity of CSCs (**Figure 4E and F**). Furthermore, silencing of Suz-12 could significantly decrease the IC_50_ value of DTX in the CSCs (**Figure 4G**). Thus, silencing of Suz-12 could mimic the effects of miR-200b on CSCs growth and chemoresistance, suggesting that Suz-12 was engaged in the regulation of CSCs induced by miR-200b.

**Figure 5:**
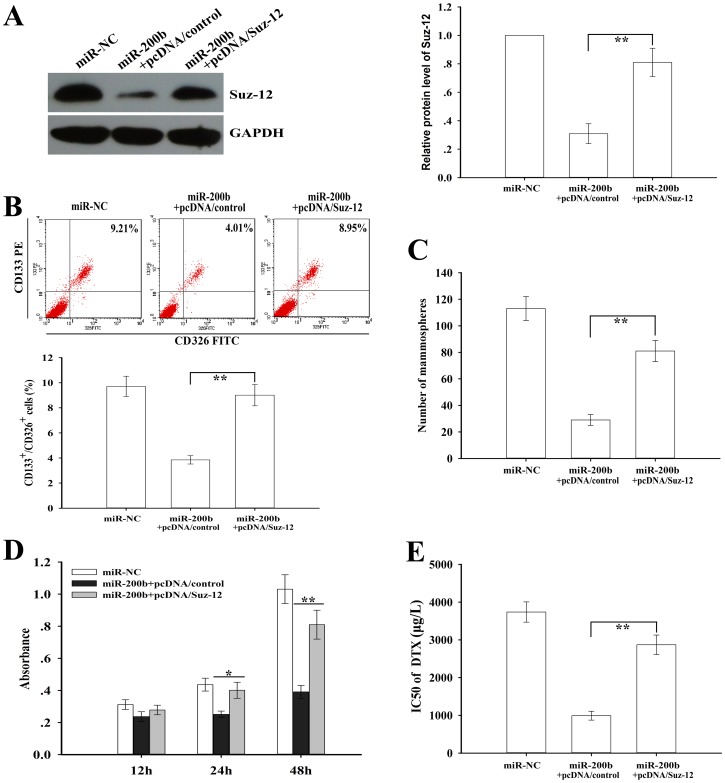
Overexpressed Suz-12 rescues the effects of enforced miR-200b expression on CSCs growth and chemoresistance. (**A**) The protein level of Suz-12 in CSCs from SPC-A1/DTX cells transfected with the indicated vectors. GAPDH was used as an internal control. (**B**) Flow cytometry analysis of percentage of CD133^+^/CD326^+^ CSCs in SPC-A1/DTX cells after transfection of the indicated vectors. (**C**) Mammosphere forming ability of SPC-A1/DTX cells (1000 cells) after transfection of the indicated vectors. (**D**) CCK-8 analysis of cell viability of the CSCs (SPC-A1/DTX) transfected with the indicated vectors at the indicated time. (**E**) Suz-12 upregulation partianlly rescued the effects of enforced miR-200b expression on reversing chemoresistance. Cell viability was measured by CCK8 assay. Data were presented as mean ± SD of at least three independent experiments. *p<0.05, **p<0.01.

To further confirm that Suz-12 was a functional target of miR-200b in the regulation of CSCs, pcDNA/Suz-12 or pcDNA/control was transfected into CSCs (SPC-A1/DTX) stably transfected with pcDNA/miR-200b or pcDNA/miR-NC. 48h after transfection, Western botting assay was performed to detect the expression of Suz-12 protein, and we showed that pcDNA/Suz-12 could reverse the decreased expression of Suz-12 in CSCs from the SPC-A1/DTX cells induced by miR-200b upregulation (**Figure 5A**). Meanwhile, overexpression of Suz-12 could not only reverse the decreased percentage of CD133^+^/CD326^+^ CSCs growth and mammosphere forming ability, but also reverse the decreased growth capacity and IC_50_ value of docetaxel in the CSCs from the SPC-A1/DTX cells induced by miR-200b upregulation (**Figure 5B-E**). Thus, overexpression of Suz-12 could reverse the effects of miR-200b upregulation on the regulation of CSCs, suggesting that Suz-12 was a functional target of miR-200b in the regulation of CSCs from the docetaxel-resistant LAD cells.

### MiR-200b inhibits CSCs by regulating Suz-12/E-cadherin signaling

**Figure 6:**
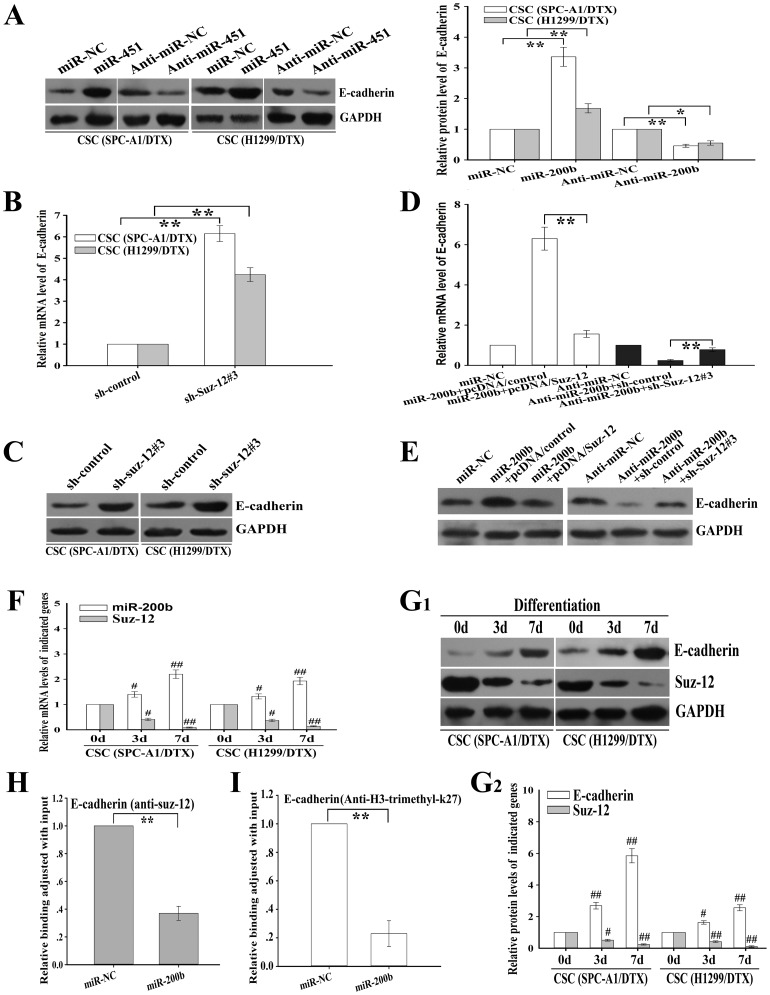
MiR-200b has effects on CSCs by regulating Suz-12/E-cadherin. (**A**) Western blotting detection of E-cadherin protein expression in the indicated cells transfected with the indicated vectors. (**B**) qRT-PCR detection of E-cadherin mRNA expression as measured by in the CSCs transfected with the indicated vectors. (**C**) Western blotting detection of E-cadherin protein expression in the CSCs transfected with the indicated vectors. (**D**) and (**E**) Suz-12 overexpression rescued the increased level of both mRNA and protein expression of E-cadherin in CSCs (SPC-A1/DTX) mediated by miR-200b upregulation, while silencing of Suz-12 rescued the decreased expression of mRNA and protein of E-cadherin in CSCs (SPC-A1/DTX) induced by miR-200b repression. (**F**) qRT-PCR detection of miR-200b and Suz-12 mRNA expression at the indicated time after plating CD133^+^/CD326^+^ CSCs under differentiation conditions. (**G_1_, G_2_**) The protein levels of Suz-12 and E-cadherin at the indicated time after cultivating CD133^+^/CD326^+^ CSCs under differentiation conditions. (**H**) MiR-200b upregulation decreased the amount of Suz-12 binding to the promoter of E-cadherin in CSCs (SPC-A1/DTX). Data were adjusted with input and determined relative to the control group. (**I**) MiR-200b overexpression reduced trimethylation of histone H3-lysine-27 (H3-K27) at E-cadherin promoter in CSCs (SPC-A1/DTX). Data were adjusted with input and determined relative to the control group. GAPDH was used as an internal control. Data were presented as mean ± SD of at least three independent experiments. *p<0.05, **p<0.01; ^#^ *p*<0.05, ^##^ *p*<0.01, compared with corresponding 0 day groups.

Recent reports have indicated that the emergence of CSCs occurs in part as a result of EMT, for example, through cues from tumor stromal components. Therefore, we will determine whether downregulation of miR-200b promotes CSCs from docetaxel-resistant LAD cells through EMT induction. Previously, miR-200b has been reported to acts as a tumor suppressor that blocks CSC formation by inhibiting the PRC2 polycomb complex, and hence preventing the repression of E-cadherin and other critical target genes. First, we analyzed the effects of miR-200b and Suz-12 on the protein expression of E-cadherin. Both overexpression of miR-200b and silencing of Suz-12 induced the increased mRNA or protein expression of E-cadherin in CSCs, while silencing of miR-200b showed the opposite effects (**Figure 6A, B and C**). Also, overexpression of Suz-12 could rescue the increased mRNA and protein expression of E-cadherin in CSCs from SPC-A1/DTX cells induced by miR-200b upregulation, while silencing of Suz-12 could rescue the decreased mRNA and protein expression of E-cadherin in CSCs from SPC-A1/DTX cells induced by miR-200b downregulation (**Fig. 6D and E**). Importantly, differentiation of CSCs into docetaxel-resistant LAD cells resulted in the repression of Suz12 and upregulation of miR-200b and E-cadherin (**Figure 6F, G** **1 and G2**). Next, we further revealed how miR-200b involved in regulation of E-cadherin by targeting Suz-12. PRC2 epigenetically silences gene transcription by generating H3K27Me3. Here, by performing ChIP assay, we showed that miR-200b decreased Suz-12 binding and H3K27Me3 at E-cadherin promoter in CSCs *in vivo* (**Figure 6H and I**). These results suggested that miR-200b regulated CSCs via downregulation of E-cadherin, at least partially by targeting Suz-12.

### HDAC1 repression enhances promoter activities of miR-200b by upregulating the histone H3-acetylation level at miR-200b promoters through a Sp1-dependent pathway

**Figure 7:**
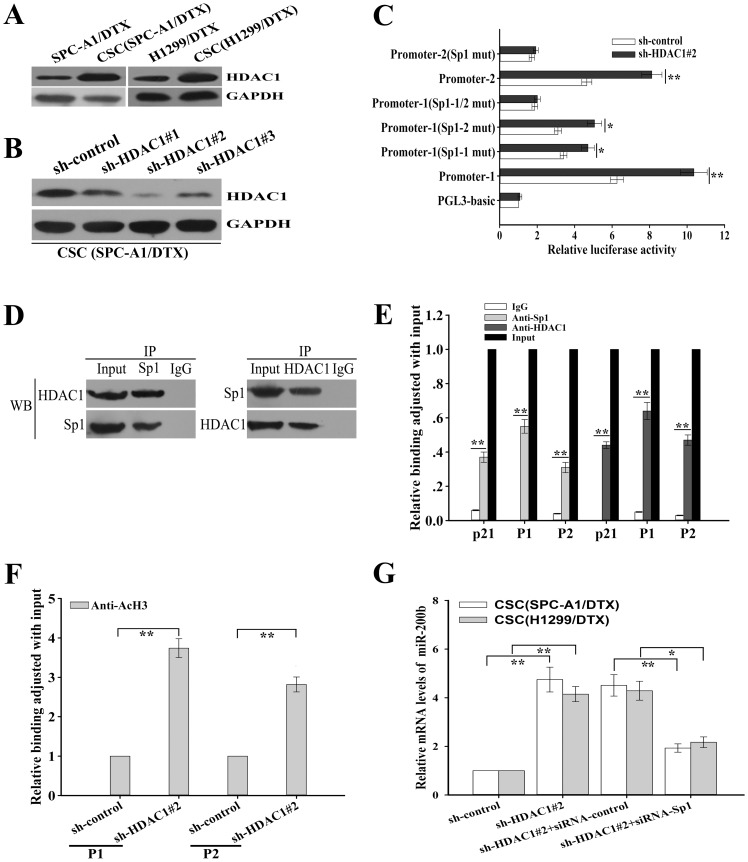
HDAC1 repression enhances miR-200b promoter activities and up-regulates the histone H3-acetylation level at the promoters through the Sp1-dependent pathway. (A) Western blotting detection of HDAC1 protein expression in CSCs from docetaxel-resistant LAD cells or parental docetaxel-resistant LAD cells. GAPDH was used as an internal control. (B) Western blotting detection of HDAC1 protein expression in CSCs from SPC-A1/DTX cells transfected with either HDAC1-shRNAs (HDAC1-shRNA#1, #2 or #3) or shRNA-control vectors, which indicated that the inhibitory effect of HDAC1-shRNA#2 was the most effective shRNA vector. GAPDH was used as an internal control. (C) Luciferase activities after transfection of the sh-HDAC1#2 or the control vectors into CSCs (SPC-A1/DTX) that had been previously cotransfected with Renilla and either wild-type, Sp1 mutant or PGL3-basic vector-firefly-promoter reporter constructs. Data was normalized to Renilla luciferase activity and determined relative to PGL3-basic-vector promoter activity. (D) The co-immunoprecipitation assay indicated that HDAC1 co-immunoprecipitated with Sp1 *in vivo* in CSCs (SPC-A1/DTX). HDAC1 and Sp1 were visualized by Western blotting (WB). (E) Chromatin immunoprecipitation (ChIP) assay indicated that HDAC1 and Sp1 bound to the miR-200b promoters *in vivo*. ChIP assays were performed in CSCs (SPC-A1/DTX) with antibodies directly against Sp1, HDAC1 or IgG control. ChIP-derived DNA was amplified after immunoprecipitation by qRT-PCR with specific primers that were designed to amplify the sequences containing the putative Sp1-binding sites. Data were shown relative to qRT-PCR products amplified with input DNA before immunoprecipitation. p21 was used as a positive control. (F) The ChIP assays indicated that HDAC1 decreased the histone H3-acetylation level at the miR-200b promoters through the Sp1-dependent pathway. ChIP assays were performed with antibody directly against acetyl-Histone H3 (AcH3) in CSCs (SPC-A1/DTX) that were transfected with shRNA-control or shRNA-HDAC1#2. ChIP-derived DNA was amplified after immunoprecipitation by qRT-PCR with primers designed to amplify the sequences containing the putative Sp1-binding sites at the miR-200b promoters. Data was normalized to qRT-PCR products that were amplified with input DNA before immunoprecipitation. (G) The mRNA level of miR-200b as measured by qRT-PCR in CSCs from docetaxel-resistant LAD cells after transfection with the sh-HDAC1#2 or the control vectors or co-transfection with siRNA-Sp1 or siRNA-control vector, which indicated that HDAC1 repression upregulated the miR-200b expression at least partially through the Sp1-dependent pathway. Data were normalized to U6 RNA. Data were presented as mean ± SD of at least three independent experiments. *p<0.05, **p<0.01.

In previous report, we have shown that upregulation of HDAC1 is one of the specific mechanisms responsible for miR-200b repression in the docetaxel-resistant LAD cells. However, whether HDAC1 mediates epigenetic regulation of miR-200b in CSCs remains unknown. Here, we showed that HDAC1 was upregulated in CSCs (**Figure 7A**). Then, HDAC1-shRNAs (HDAC1-shRNA1, 2 or 3) were designed and transfected into CSCs (SPC-A1/DTX) and the inhibitory effect of HDAC1-shRNA2 was the most effective shRNA (**Figure 7B**). Moreover, downregulation of HDAC1 significantly enhanced promoter activities of miR-200b in CSCs through a Sp1-dependent pathway (**Figure 7C**). Co-immunoprecipitation and ChIP assay indicated that both HDAC1 and Sp1 could bind to the miR-200b promoters *in vivo* (**Figure 7D and E**). Next, the HDAC1-shRNA2 or control-shRNA vector was transfected into the CSCs (SPC-A1/DTX) and the transfected cells were used for ChIP assay (**Figure 7F**). Then, the ChIP-derived DNA immunoprecipitated from anti-acetyl-Histone H3 antibody was amplified with the primers that were designed to amplify the sequences containing the Sp1 binding sites at the promoters of miR-200b, which indicated that silencing of HDAC1 could upregulate the histone H3-acetylation level at the miR-200b promoters through the Sp1-dependent pathway (**Figure 7F**). Furthermore, qRT-PCR assay indicated that silencing of HDAC1 significantly elevated the expression level of miR-200b in CSCs partially in the Sp1-dependent manner (**Figure 7G**). Taken together, HDAC1 might be an important regulator of promoter activities of miR-200b by upregulating the histone H3-acetylation level at miR-200b promoters through the Sp1-dependent pathway.

### HDAC1 repression-mediated miR-200b expression inhibits CSCs formation and reverses chemoresistance of CSCs by regulating Suz-12/E-cadherin

**Figure 8:**
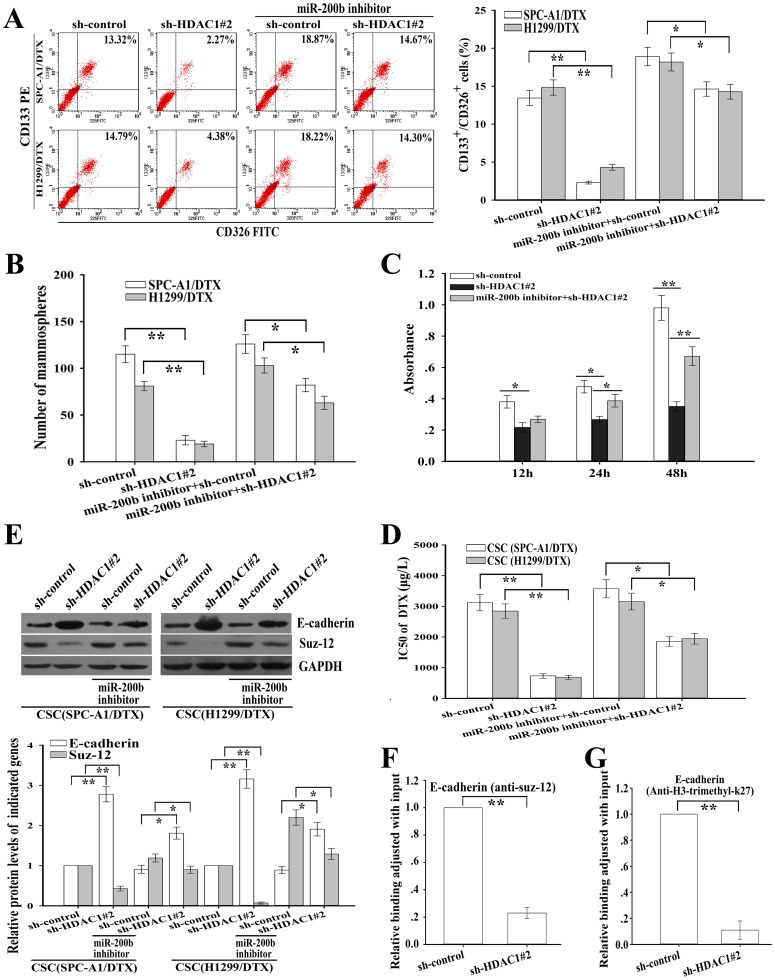
HDAC1 suppression-mediated miR-200b expression inhibits CSCs growth and reverses chemoresistance of CSCs by regulating Suz12/E-cadherin signaling. (A) Flow cytometry analysis of percentage of CD133^+^/CD326^+^ CSCs in docetaxel-resistant LAD cells after transfection of the indicated vectors. (B) Mammosphere forming ability of docetaxel-resistant LAD cells (1000 cells) after transfection of the indicated vectors. (C) Cell viability as measured by CCK-8 assay of the CSCs (SPC-A1/DTX) transfected with the indicated vectors at the indicated time. (D) MiR-200b inhibition rescued the effects of HDAC1 repression on reversing chemoresistance. Cell viability was determined by CCK8 assay. (E) Repression of HDAC1 downregulated Suz-12 expression and upregulated E-cadherin expression in CSCs from docetaxel-resistant LAD cells partially in a miR-200b-dependent manner. GAPDH was used as an internal control. (F) Suppression of HDAC1 decreased the amount of Suz-12 binding to the promoter of E-cadherin in CSCs (SPC-A1/DTX). Data were adjusted with input and determined relative to the control group. (G) HDAC1 repression reduced trimethylation of H3-K27 at E-cadherin promoter in CSCs (SPC-A1/DTX). Data were adjusted with input and determined relative to the control group. Data were presented as mean ± SD of at least three independent experiments. *p<0.05, **p<0.01.

Next, we focus on whether HDAC1 repression-mediated miR-200b expression has effects on CSCs formation and chemoresistance. And, HDAC1 repression significantly reduced the percentage of CD133^+^/CD326^+^ CSCs and suppressed mammosphere forming ability in the docetaxel-resistant LAD cells (**Figure 8A and B**). Likewise, HDAC1 repression could significantly inhibit growth and reverse chemoresistance of CSCs (**Figure 8C and D**). Intriguingly, the effects of HDAC1 repression on CSCs growth and chemoresistance were partially abrogated by miR-200b inhibition (**Figure 8A–D**). Then, we further analyzed the effects of HDAC1 repression-mediated miR-200b on Suz-12/E-cadherin signaling in CSCs. As shown in **figure 8E**, repression of HDAC1 significantly reduced Suz-12 expression and increased E-cadherin expression while the effects were partially abrogated by miR-200b inhibition. Also, we showed that inhibition of HDAC1 could significantly decrease Suz-12 binding and H3K27Me3 at E-cadherin promoter in CSCs *in vivo* (**Figure 8F and G**). Thus, the HDAC1/miR-200b/Suz-12-E-cadherin signaling might be responsible for regulating growth and chemoresistance of CSCs derived from the docetaxel-resistant LAD cells.

### HDAC1 repression or miR-200b upregulation reduces tumorigenicity and reverses chemoresistance of CSCs *in vivo*

**Figure 9:**
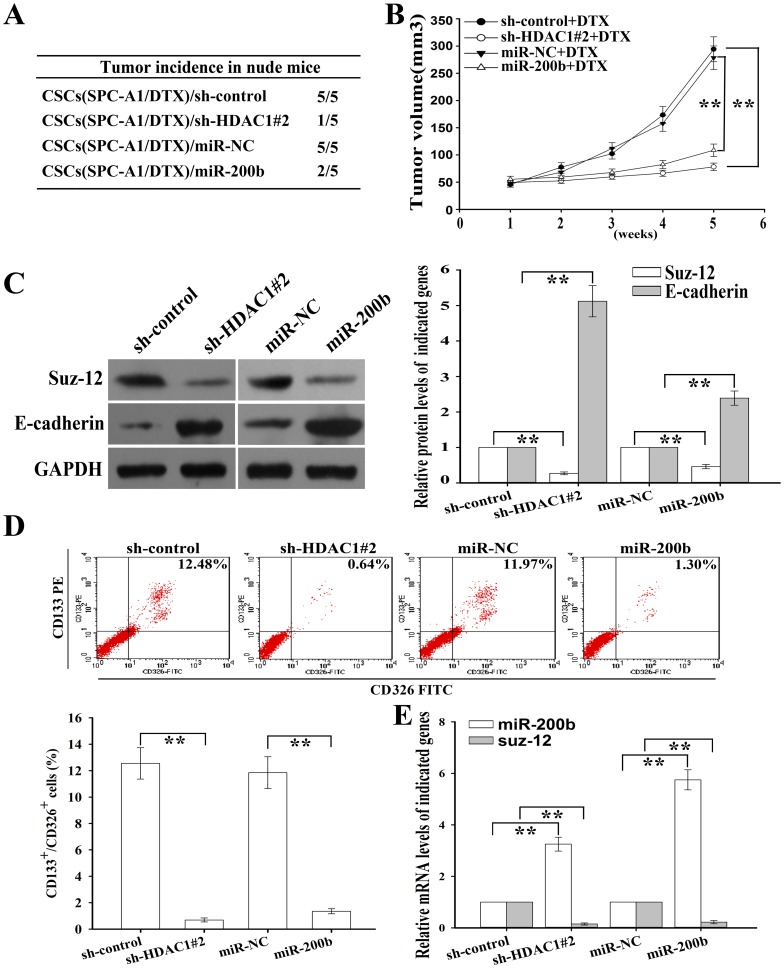
HDAC1 repression or miR-200b overexpression reduces tumorigenicity and reverses chemoresistance of CSCs *in vivo*. (**A**) Tumorigenicity in nude mice subcutaneously injected with CSCs from SPC-A1/DTX cells (5000 cells/mouse, n = 5) that were stably transfected with the indicated vectors previously. (**B**) Tumor volume in nude mice injected with H1299/DTX cells that were stably transfected with the indicated vectors and were combined with DTX treatment. Data were presented as mean ± SD. (**C**) The protein level of E-cadherin and Suz-12 in tumors of the indicated groups that were provided at 5 weeks after the inoculation. GAPDH was used as an internal control. (**D**) Flow cytometry detection of percentage of CD133^+^/CD326^+^ CSCs in tumors of the indicated groups. (**E**) The mRNA level of miR-200b and Suz-12 in tumors of the indicated groups. Data were normalized to U6 RNA and GAPDH, respectively. Data were presented as mean ± SD of at least three independent experiments. **p<0.01.

To explore the effects of HDAC1/miR-200b expression on CSCs in *vivo*, approximately 1.0×10^3^ CSCs from SPC-A1/DTX cells stably transfected with pSil/HDAC1-shRNA, pcDNA/miR-200b or their corresponding control vectors, were subcutaneously transplanted into nude mice. As shown in **Figure 9A**, transfection of CSCs with HDAC1-shRNA or pcDNA/miR-200b vector could obviously block tumor initiation in nude mice. Then, we further analyzed the effects of HDAC1 and miR-200b on *in vivo* chemoresistance of docetaxel-resistant LAD cells, and showed that H1299/DTX cells stably transfected with HDAC1-shRNA or pcDNA/miR-200b grew significantly more slowly than those stably transfected with the corresponding control groups while combined with DTX treatment, which indicated that silencing of HDAC1 or overexpression of miR-200b could significantly increase the *in vivo* chemosensitivity of CSCs to DTX (**Figure 9B**). Also, our data showed that Suz-12 was down-regulated while E-cadherin and miR-200b were up-regulated in HDAC1-shRNA and miR-200b-expression groups compared with those in the corresponding control groups (**Figure 9C and E**). Also, the percentage of CD133^+^/CD326^+^ CSCs was decreased in HDAC1-shRNA or pcDNA/miR-200b-transfected groups than that in the corresponding control groups (**Figure 9D**). These data suggested that dysregulation of HDAC1/miR-200b could promote CSCs growth and chemoresistance *in vivo*.

### Suz-12, up-regulated in docetaxel-insensitive LAD tissues, is inversely correlated with miR-200b and positively correlated with HDAC1

Our previous work has shown that miR-200b repression is linked to the chemoresistance of the clinical LAD tissues [19]. To further understand the clinical significance of the present work, 49 cases of clinical LAD tissues were gathered from patients at advanced stage and divided into “sensitive” and “insensitive” groups basing on the responses to the docetaxel-based chemotherapy, and then qRT-PCR assay was used to detect the expression of HDAC1, miR-200b and Suz-12. The results indicated that the mean expression level of Suz-12 mRNA in docetaxel-insensitive LAD tissues was significantly higher than that in docetaxel-sensitive LAD tissues (*p*<0.01; **Figure S1A**). Meanwhile, the mean expression level of HDAC1 mRNA in docetaxel-insensitive LAD tissues was significantly higher compared with that in docetaxel-sensitive tissues (*p*<0.05; **Figure S1B**). Furthermore, the levels of Suz-12 was inversely correlated with miR-200b and positively correlated with HDAC1 while miR-200b was inversely correlated with HDAC1 in 49 LAD tissues as determined by linear regression analysis (Figure S1C-E). These results further indicated that dysregulation of HDAC1/miR-200b/Suz-12 pathway is correlated with responses of LAD patients to docetaxel-based chemotherapy.

## Discussion

The presence of CSCs has already been associated with chemotherapeutic failure in a variety of solid tumors including LAD. CSCs are always resistant to the standard chemotherapies that reduce LAD mass instead of successful eliminating CSCs which then cause a relapse of LAD. Thus, revealing the underlying molecular mechanisms responsible for maintenance and chemoresistance of CSCs has become a crucial issue in clinical treatment of human LAD.

CSC properties in tumor cells can be determined according to the ability to form tumors at limiting dilutions *in vivo*. Also, CSCs exhibit several key characteristics *in vitro*, for instance, CSCs are able to form mammospheres in suspension culture, undergo differentiation while under differentiation conditions and can be identified by expression of cell-surface markers [22]–[24]. Furthermore, recent studies have shown that the cell-surface markers CD133 and EpCAM (CD326) are regarded as cancer stem-cell-related markers for many types of cancers including lung cancer [3], [22], [25], [26]. In this study, the CD133^+^/CD326^+^ cells are isolated from the docetaxel-resistant LAD cells by CD133 and CD326 MicroBeads and have high capability of self-renewal and can differentiate back into the parental cells under differentiation conditions *in vitro*. Also, the CD133^+^/CD326^+^ cells express significantly higher levels of several well-known stemness markers such as Sox-2, Oct-4, Suz-12, and Bmi-1 than the corresponding parental cells [17], [27]–[29]. More importantly, the CD133^+^/CD326^+^ cells possess higher capacity of tumorigenicity *in vivo*. Thus, basing on these data, we conclude that the CD133^+^/CD326^+^ subpopulation cells exhibit key properties of CSCs.

Recent studies have highlighted that miRNAs including miR-200b have been firmly linked with chemoresistance of CSCs in many types of human tumors [30], [31]. The miR-200 families target the multiple sites in the 3′UTRs of ZEB1 and ZEB2, which then regulate expression of E-cadherin [30], [32]. Here, we showed that re-expression of miR-200b could reduce CSCs growth and reverse chemoresistance of CSCs both *in vitro* and *in vivo*. Emerging evidences have associated E-cadherin level with the CSC states that are responsible for exhibiting an increased resistance to chemotherapy [33], [34]. Enforced miR-200b expression mediates the upregulation of E-cadherin by directly inhibiting its targets such as ZEB1, ZEB2 and Suz-12. Suz-12 protein is a member of PRC2 maintaining CSCs self-renewal and pluripotency, which is responsible for aberrant silencing of tumor suppressor genes that are always linked to the aggressiveness of the tumors [35]–[38]. Suz-12 can bind into the promoter of E-cadherin and mediate E-cadherin repression by H3K27Me3, which is responsible for the maintenance of the CSC phenotypes. Previously, Suz-12 has been confirmed as a perfectly homologous and highly conserved target gene of miR-200b in CSCs from transformed breast epithelial cells (MCF-10A). Herein, Suz-12 is also identified as a functional target of miR-200b in the CSCs derived from the docetaxel-resistant LAD cells. Furthermore, Suz-12 repression can phenocopy the effects of miR-200b on CSCs.

Increasing evidence has shown that miR200b/Suz-12/E-cadherin pathway is involved in the regulation of CSCs maintenance, tumorigenicity and chemoresistance. First, shRNA-mediated Suz-12 repression significantly suppresses CSCs self-renewal, tumorigenicity and growth and reverses the chemoresistance of CSCs. Second, silencing of Suz-12 significantly elevates the protein and mRNA levels of E-cadherin in the CSCs. Third, Suz-12 is upregulated while miR-200b and E-cadherin are downregulated in CSCs, and CSCs differentiate back into docetaxel-resistant LAD cells which result in the repression of Suz-12 and upregulation of miR-200b and E-cadherin. Fourth, enforced miR-200b expression significantly represses CSCs self-renewal, tumorigenicity and growth. Additionally, the effects of overexpressed miR-200b on CSCs self-renewal and growth could be partially rescued by Suz-12 overexpression. Fifth, enforced miR-200b expression significantly decreases Suz-12 expression and increases E-cadherin expression, while miR-200b repression has opposite effects. Moreover, the effects of upregulation (or downregulation) of miR-200b on E-cadherin expression are partially rescued by upregulation (or downregulation) of Suz-12. Finally, ectopic miR-200b expression decreases Suz-12 binding and H3K27Me3 at the E-cadherin promoter in CSCs *in vivo*. Therefore, the miR-200b/Suz-12/E-cadherin pathway is responsible for maintenance, tumorigenicity and chemoresistance of CSCs.

Although miR-200b repression has been a crucial issue in maintenance, tumorigenicity and chemoresistance of CSCs, the molecular mechanisms responsible for miR-200b repression in CSCs remain unclear and need to be further elucidated. As an important epigenetic mechanism, histone acetylation has been engaged in many types of cancers by transcriptional regulation of genes expression such as miR-200a, miR-449 and miR-15a [39]–[41]. Histone acetylation is regulated by the histone acetyl transferases and HDACs. HDACs involve in epigenetic regulation of gene expression through removing acetyl groups from lysine residues around gene promoters. HDACs have been engaged in regulation of growth, aggressiveness and self-renewal of CSCs, which indicates that they are promising therapeutic targets [42], [43]. Basing on phylogenetic analysis, sequence homology and function, HDACs can be divided into four distinct classes: class I (HDAC1, 2, 3 and 8), class II (HDAC4, 5, 6, 7, 9 and 10), class III (SIRT 1–7) and class IV (HDAC11) [44]. Previously, we have reported that HDAC1/4 is the specific regulator responsible for silencing of miR-200b in the docetaxel-resistant LAD cells. Generally, HDACs are lack of DNA binding domain and can’t bind to DNA directly and they interact with DNA through the specific proteins. Here, we show that HDAC1 can interact with Sp1 *in vivo*, which is consistent with the previous study [45]. Moreover, our data indicate that both HDAC1 and Sp1 can co-localize to the two promoters of the miR-200b *in vivo*. Also, HDAC1 repression up-regulates the histone H3-acetylation level at the miR-200b promoters through the Sp1-dependent pathway. Importantly, HDAC1 is up-regulated in CSCs and downregulation of HDAC1 can significantly increase the level of miR-200b expression in CSCs. Therefore, HDAC1 mediates miR-200b repression in CSCs through the Sp1-dependent pathway.

Generally, HDACs repression activates gene transcription through forming an open chromatin structure. However, HDAC inhibition can also induce repressive histone modifications through recruitment of corepressor complex such as pRB to the promoter region [46]. Additionally, an antitumor dose of HDAC inhibitors can only have effects on less than 10% of expressed genes in the given malignant cells [47]. Here, HDAC1 repression significantly reduces Suz-12 expression, which may be due to the reasons as follows: HDAC1 reduces Suz-12 by recruitment of the corepressor complex to the promoter region of Suz-12. Actually, HDAC1 may not the specific HDACs accounting for histone modifications at the promoter region of Suz-12 and HDAC1 decreases Suz-12 expression by upregulating the certain miRNAs including miR-200b which then target at the 3′-UTR of Suz-12 mRNA. Also, HDAC1 inhibition significantly decreases Suz-12 binding and H3K27Me3 at E-cadherin promoter *in vivo,* which then mediates upregulation of E-cadherin expression in CSCs. Furthermore, HDAC1 repression significantly reduces the CSCs growth and self-renewal and reverses chemoresistance of CSCs in a miR-200b-dependent manner. Taken together, HDAC1 repression-mediated overexpression of miR-200b inhibits maintenance of CSCs and reverses chemoresistance of CSCs both *in vitro* and *in vivo* by regulating Suz-12/E-cadherin signaling. Furthermore, our clinical data show that HDAC1 is inversely correlated with miR-200b and positively correlated with Suz-12 in clinical LAD tissues.

Taken together, the novel HDAC1/miR-200b/Suz-12/E-cadherin pathway may play essential roles in regulating maintenance, tumorigenicity and chemoresistance of CSCs in human LAD. This work will provide a novel strategy for reversing chemoresistance of human LAD.

## Supporting Information

Figure S1 **Suz-12 is inversely correlated with miR-200b, positively correlated with HDAC1 and up-regulated in docetaxel-insensitive human LAD tissues.** (A) The relative mRNA level of Suz-12 was determined in docetaxel-sensitive (n = 21) and insensitive (n = 28) human LAD tissues by qRT-PCR and normalized to GAPDH RNA. **P<0.01. (B) The relative mRNA level of HDAC1 was determined in docetaxel-sensitive (n = 21) and insensitive (n = 28) human LAD tissues by qRT-PCR and normalized to GAPDH RNA. *p<0.05. (C) The mRNA levels of Suz-12 and miR-200b were inversely correlated in 49 LAD tissues as determined by linear regression analysis. The Suz-12 level was normalized to GAPDH RNA while miR-200b was normalized to U6 RNA. Spearman rank test rho and *P* values (2-tailed) were shown. (D) The mRNA levels of miR-200b and HDAC1 were inversely correlated in 49 LAD tissues as determined by linear regression analysis. The HDAC1 level was normalized to GAPDH while miR-200b was normalized to U6 RNA. Spearman rank test rho and *P* values (2-tailed) were shown. (E) The mRNA levels of Suz-12 and HDAC1 were positively correlated in 49 LAD tissues as determined by linear regression analysis. The mRNA level was normalized to GAPDH RNA. Data were presented as mean ± SD of at least three independent experiments. *p<0.05, **p<0.01.
(TIF)

Table S1 **Primers for promoter and gene expression experiments**
(DOC)

Table S2 **Primers for sh-RNA experiments.**
(DOC)

Table S3 **Primers for real-time quantitative PCR.**
(DOC)

Table S4 **Primers for ChIP-PCR.**
(DOC)
