# Supplementary figures and images for: Histone Deacetylase 1/Sp1/MicroRNA-200b Signaling Accounts for Maintenance of Cancer Stem-Like Cells in Human Lung Adenocarcinoma
Source: PLoS One. 2014 Oct 3;9(10):e109578. doi: 10.1371/journal.pone.0109578 (PMC4184862; doi:10.1371/journal.pone.0109578)

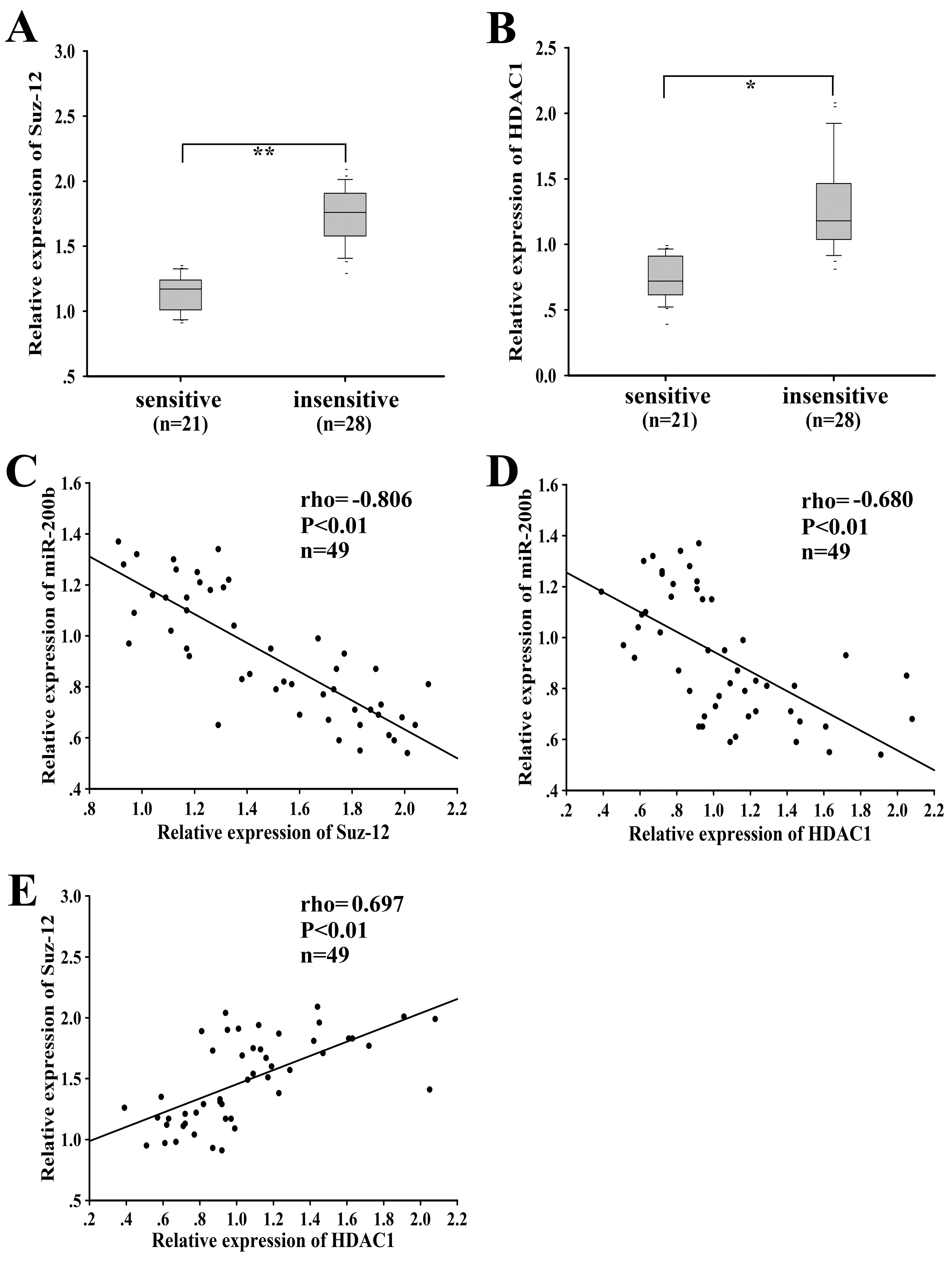

Supplement: Figure S1 — Suz-12 is inversely correlated with miR-200b, positively correlated with HDAC1 and up-regulated in docetaxel-insensitive human LAD tissues. (A) The relative mRNA level of Suz-12 was determined in docetaxel-sensitive (n = 21) and insensitive (n = 28) human LAD tissues by qRT-PCR and normalized to GAPDH RNA. **P<0.01. (B) The relative mRNA level of HDAC1 was determined in docetaxel-sensitive (n = 21) and insensitive (n = 28) human LAD tissues by qRT-PCR and normalized to GAPDH RNA. *p<0.05. (C) The mRNA levels of Suz-12 and miR-200b were inversely correlated in 49 LAD tissues as determined by linear regression analysis. The Suz-12 level was normalized to GAPDH RNA while miR-200b was normalized to U6 RNA. Spearman rank test rho and P values (2-tailed) were shown. (D) The mRNA levels of miR-200b and HDAC1 were inversely correlated in 49 LAD tissues as determined by linear regression analysis. The HDAC1 level was normalized to GAPDH while miR-200b was normalized to U6 RNA. Spearman rank test rho and P values (2-tailed) were shown. (E) The mRNA levels of Suz-12 and HDAC1 were positively correlated in 49 LAD tissues as determined by linear regression analysis. The mRNA level was normalized to GAPDH RNA. Data were presented as mean ± SD of at least three independent experiments. *p<0.05, **p<0.01. (TIF) [file pone.0109578.s001.tif]
