# Supplementary material for: Histone Deacetylase 1/Sp1/MicroRNA-200b Signaling Accounts for Maintenance of Cancer Stem-Like Cells in Human Lung Adenocarcinoma
Source: PLoS One. 2014 Oct 3;9(10):e109578. doi: 10.1371/journal.pone.0109578 (PMC4184862; doi:10.1371/journal.pone.0109578)
Supplement: Table S1 — Primers for promoter and gene expression experiments (DOC) [file pone.0109578.s002.doc]

**Supplementary Table S1: Primers for promoter and gene expression experiments**

| **Name primer sequences** |
| --- |
| **miR200b Promoter-1 F** 5’-CGGGGTACCCAGAGGTGGAGAGGCGAGAG-3’ |
| **R**  5’-CCCAAGCTTGGGGCCTCGGGAGGGAAGAGC-3’ |
| **miR200b Promoter-2 F** 5’-CGGGGTACCTTTACAGCCCGGATCACTGG-3’ |
| **R**  5’-CCCAAGCTTCGCTTTCTTGTCAACCGTCG-3’ |
| **miR200b promoter-1,Sp1-1 F**  5’-CGGCAAGGTGGGTTCGGGACGGAGTCTGCG-3’ |
| **R** 5’-CGTCCCGAACCCACCTTGCCGCCCACCGAG-3’ |
| **miR200b promoter-1,Sp1-2 F**  5’-CCTGTGTGGTTCGGGGAGCACTGCTCCTTG-3’ |
| **R**  5’-TGCTCCCCGAACCACACAGGTGCGAGCTCC-3’ |
| **miR200b promoter-2, Sp1 F**  5’-TGTTCTCTGTGGTTCGGGTGGACGTGGCCCGGAC-3’ |
| **R** 5’-TCCACCCGAACCACAGAGAACACACCAGCTCCT -3’ |
| **pLUC/Suz-12/3’UTR-wt F** 5’- CCGCTCGAGTGAGGCTATTTAACGAATAGTGTGGAT -3’ |
| **R** 5’-ATTTGCGGCCGCCCTATCATCTAGAGCTCGCAAATAG -3’ |
| **pLUC/Suz-12/3’UTR-mut F**  5’- TCTGATCTGTATCTTACGAAGTTCTTAGTCATTGA-3’ |
| **R** 5’-GTAGACATAGAATGCTAGTCAAGAATCAGTAACTAAA-3 |
| **Suz-12 expression F** 5’-CGGGGTACCCTGAGACACTATCTGTTTCCAAAG -3’ |
| **R** 5’-CCCAAGCTTTACAGACGATTGTGGCCACTACTA -3’ |
