# Supplementary material for: Histone Deacetylase 1/Sp1/MicroRNA-200b Signaling Accounts for Maintenance of Cancer Stem-Like Cells in Human Lung Adenocarcinoma
Source: PLoS One. 2014 Oct 3;9(10):e109578. doi: 10.1371/journal.pone.0109578 (PMC4184862; doi:10.1371/journal.pone.0109578)
Supplement: Table S2 — Primers for sh-RNA experiments. (DOC) [file pone.0109578.s003.doc]

**Supplementary Table S 2: Primers for sh-RNA experiments**

**Name primer sequences**

**sh-Suz-12 #1 F** 5’- CCGGGCTGACAATCAAATGAATCAT CTCGAGATGATTCATTTGATTGTCAGCTTTTTG -3’

**R** 5’- AATTCAAAAA GCTGACAATCAAATGAATCATCTCGAG ATGATTCATTTGATTGTCAGC -3’

**sh- Suz-12 #2 F** 5’- CCGGCCAAACCTCTTGCCACTAGAACTCGAGTTCTAGTGGCAAGAGGTTTGGTTTTTG -3

**R** 5’- AATTCAAAAACCAAACCTCTTGCCACTAGAACTCGAGTTCTAGTGGCAAGAGGTTTGG -3’

**sh- Suz-12 #3 F** 5’- CCGGGCTTACGTTTACTGGTTTCTTCTCGAG AAGAAACCAGTAAACGTAAGCTTTTTG -3’

**R**5’-AATTCAAAAAGCTTACGTTTACTGGTTTCTTCTCGAGAAGAAACCAGTAAACGTAAGC -3’

**sh-control F** 5’- CCGGGCTTCTCCGAACGTGTCACGTCTCGAG AAGAAACCAGTAAACGTAAGCTTTTTG -3’

**R** 5’- AATTCAAAAAGCTTCTCCGAACGTGTCACGTCTCGAGAAGAAACCAGTAAACGTAAGC -3’

**sh-HDAC1 #1 F** 5’-CACCGCTCCATCCGTCCAGATAACATTCAAGAGATGTTATCTGGACGGATGGAGCTTTTTTG-3’

**R** 5’-GATCCAAAAAAGCTCCATCCGTCCAGATAACATCTCTTGAATGTTATCTGGACGGATGGAGC -3’

**sh-HDAC1 #2 F** 5’-CACCGGAGAGTACTTCCCAGGAACTTTCAAGAGAAGTTCCTGGGAAGTACTCTCCTTTTTTG-3

**R** 5’-GATCCAAAAAAGGAGAGTACTTCCCAGGAACTTCTCTTGAAAGTTCCTGGGAAGTACTCTCC-3’

**sh-HDAC1 #3 F** 5’-CACCGGTGAGGACTGTCCAGTATTCTTCAAGAGAGAATACTGGACAGTCCTCACCTTTTTTG-3’

**R** 5’-GATCCAAAAAAGGTGAGGACTGTCCAGTATTCTCTCTTGAAGAATACTGGACAGTCCTCACC-3’

**sh-control F** 5’-CACCGTTCTCCGAACGTGTCACGTTTCAAGAGAACGTGACACGTTCGGAGAATTTTTTG-3’

**R** 5’-GATCCAAAAAATTCTCCGAACGTGTCACGTTCTCTTGAAACGTGACACGTTCGGAGAAC -3’
