# Supplementary material for: Histone Deacetylase 1/Sp1/MicroRNA-200b Signaling Accounts for Maintenance of Cancer Stem-Like Cells in Human Lung Adenocarcinoma
Source: PLoS One. 2014 Oct 3;9(10):e109578. doi: 10.1371/journal.pone.0109578 (PMC4184862; doi:10.1371/journal.pone.0109578)
Supplement: Table S3 — Primers for real-time quantitative PCR. (DOC) [file pone.0109578.s004.doc]

**Supplementary Table S 3: Primers for real-time quantitative PCR**

**Name primer sequences**

**miR-200b Stem-loop** 5’-GTCGTATCCAGTGCAGGGTCCGAGGTATTCGCACTGGATACGACTCATCA-3'.

**F** 5'-GTGGAGGGTCCGAGGTATTC-3'

**R** 5'-CGTAATACTGCCTGGTAATGATG-3'

**U6 F**  5’-CTCGCTTCGGCAGCACA-3'

**R** 5’-AACGCTTCACGAATTTGCGT-3'

**Suz-12 F** 5'-GATAAAAACAGGCGCTTACAGCTT -3'

**R**  5'-AGGTCCCTGAGAAAATGTTTCGA-3'

**Bmi-1 F**  5’-AATCTAAGGAGGAGGTGA-3’

**R** 5’-AAACAAGAAGAGGTGGA-3’

**E-cadherin F** 5’- GGAGGAGAGCGGTGGTCAAA -3’

**R** 5’- TGTGCAGCTGGCTCAAGTCAA -3’

**Oct-4**  **F** 5’- CGCAAGCCCTCATTTCAC-3’

**R** 5’- CATCACCTCCACCACCTG-3’

**SOX-2 F**  5’-CCCACCTACAGCATGTCCTACTC-3'

**R** 5’-TGGAGTGGGAGGAAGAGGTAAC-3'

**GAPDH F** 5'-TGGGTGTGAACCATGAGAAGT-3'

**R**  5'-TGAGTCCTTCCACGATACCAA-3'
