# Supplementary material for: Histone Deacetylase 1/Sp1/MicroRNA-200b Signaling Accounts for Maintenance of Cancer Stem-Like Cells in Human Lung Adenocarcinoma
Source: PLoS One. 2014 Oct 3;9(10):e109578. doi: 10.1371/journal.pone.0109578 (PMC4184862; doi:10.1371/journal.pone.0109578)
Supplement: Table S4 — Primers for ChIP-PCR. (DOC) [file pone.0109578.s005.doc]

**Supplementary Table S 4: Primers for ChIP-PCR**

| **Name primer sequences** |
| --- |
| **miR200b Promoter-1 F**  5’-GGCTCGCCTTACAAGGAGCAGT-3’ |
| **R**  5’-TTTTGTTCAACCTCGGTGGGC-3’ |
| **miR200b Promoter-2 F** 5’-GGCGTGGATTTGCAGCCCTC-3’ |
| **R**  5’-GCGAAACGAGTTTGTCAGAACA-3’ |
| **P21 F**  5’-GCAGCTGAGCCTGGCCGAGTTC-3’ |
| **R** 5’-GCACCAACGCAGGCGAGGGACT-3’ |
| **E-cadherin F**  5’-ACCTCCTCCGACCTCACTTT-3’ |
| **R**  5’-AGAGGGGCATCCGTAGAAAT-3’ |
